# Spikes in the data: biological validation and applications of an assay for detecting faecal glucocorticoid metabolites in Western European hedgehogs (*Erinaceus europaeus*)

**DOI:** 10.1093/conphys/coag063

**Published:** 2026-08-24

**Authors:** Emily Harper, Kelly Yarnell, Rupert Palme, Dawn M Scott, Iain Barber, Richard W Yarnell

**Affiliations:** School of Animal, Rural & Environmental Sciences, Nottingham Trent University, Brackenhurst, Southwell, Nottinghamshire NG25 0QF, UK; School of Animal, Rural & Environmental Sciences, Nottingham Trent University, Brackenhurst, Southwell, Nottinghamshire NG25 0QF, UK; Department of Biological Sciences and Pathobiology, University of Veterinary Medicine, Vienna; School of Animal, Rural & Environmental Sciences, Nottingham Trent University, Brackenhurst, Southwell, Nottinghamshire NG25 0QF, UK; Department of Life Sciences, Aberystwyth University, Reception, Penglais, Aberystwyth, Ceredigion, SY23 3FL, UK; School of Animal, Rural & Environmental Sciences, Nottingham Trent University, Brackenhurst, Southwell, Nottinghamshire NG25 0QF, UK

**Keywords:** Biological validation, conservation, immunoassay, Life on Land, non-invasive, stress, wildlife

## Abstract

Assessment of wildlife health and welfare is an increasingly important tool in identifying environmental stressors affecting species of conservation concern. An improved understanding of physiological responses to stress can inform management practises and provide methods for detecting factors that may influence individual and population viability. Faecal glucocorticoid metabolites (FGMs) are a popular non-invasive proxy for stress. To measure FGMs, the chosen assay must be sensitive to changes, and biological validation is required to demonstrate that these changes are relevant responses to stress. This study carried out a preliminary validation of three enzyme immunoassays (EIAs) previously used on mammals, and biologically validated the most sensitive assay for use in Western European hedgehogs (*Erinaceus europaeus*), which are classified as Near Threatened (International Union for Conservation of Nature) and are a species of European conservation concern. All three assays were sensitive to FGM changes, with the 11β-hydroxyetiocholanolone EIA (hereafter, ‘assay 69a’) showing the greatest increase above baseline, and therefore being the most sensitive. A biological validation using assay 69a compared FGMs from hedgehogs exposed to stressors after admission to rehabilitation (Day 1) with FGMs collected 7 days later (Day 7) following veterinary care. Individual FGMs were highest on Day 1 (x- = 743 ng/g, standard deviation [SD] = 602.7) and reduced by Day 7 (x- = 224 ng/g, SD = 207.5). Hedgehogs with FGMs below 1000 ng/g had an 82% chance of surviving rehabilitation; at 3000 ng/g, they had a 52% chance of survival, while survival rates for those with FGMs over 10 000 ng/g were less than 1%. Finally, a comparison of wild hedgehogs with and without the parasite *Capillaria* showed higher FGMs in infected individuals, demonstrating the utility of assay 69a for measuring different stressors in Western European hedgehogs. We recommend that assay 69a be used in future hedgehog conservation and welfare studies, to better understand the environmental, and anthropogenic stressors that may contribute to their ongoing decline.

## Graphical Abstract

**Graphical Abstract:**
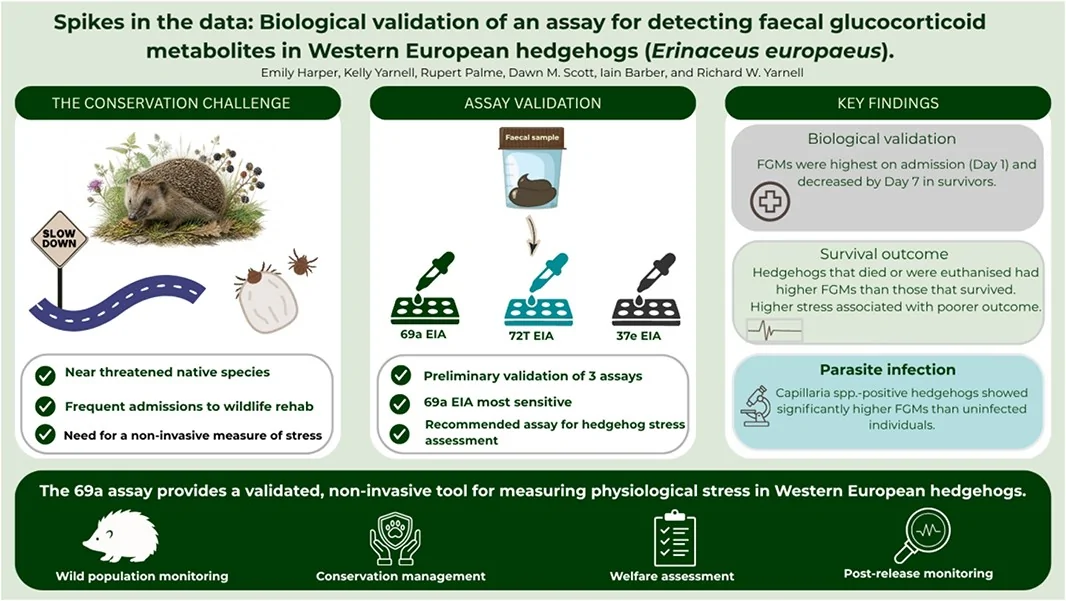

## Abbreviations

EIA, enzyme immunoassay
FGMs, Faecal glucocorticoid metabolites
HPA axis, Hypothalamic-pituitary-adrenal axis

## Introduction

The Western European hedgehog (*Erinaceus europaeus*) (hereafter, ‘hedgehog’) is classified as Near Threatened (Gazzard and Rasmussen, 2024), with populations declining across their range. The causes of the decline are not well understood, but it is hypothesized that a suite of environmental and anthropogenic stressors is likely to impact individuals, and influence wider population processes (Yarnell and Pettett, 2020). The environmental stressors include low food resource availability (Hof and Bright, 2016; Williams *et al.*, 2018), disease outbreaks (Sauer *et al.*, 2025), and, in areas where supplementary feeding is practised, an increase in conspecific encounters leading to potentially stressful agonistic behaviours (fighting) (Scott *et al.*, 2023). Anthropogenic stressors include potential disruption from noise and light in suburban settings (Berger, Barthel *et al.*, 2020; Berger, Lozano *et al.*, 2020; Finch *et al.*, 2020), as well as widespread supplementary feeding of nutritionally unsuitable food types, which pose risks to individual physiological condition (Gimmel *et al.*, 2021; Bragg *et al.*, 2024). Collectively, these stressors may impact the welfare of wild hedgehogs in varying contexts, compromising their overall fitness. Population-level welfare assessments may advance our understanding of resource differences between habitats, and associated stressors, which may help explain variable demographic rates, as well as the effects of stressors associated with variable predator density (McCleery, 2009; Lowry *et al.*, 2013; Rodewald and Gehrt, 2014; Hitchcock *et al.*, 2025). Consequently, being able to assess the relative stress levels of individual in different contexts would provide insights into which stressors have the greatest influence on welfare. The application of tools to assess environmental and anthropogenic stressors on hedgehog populations is likely to be beneficial for understanding the drivers of decline, and enabling targeted conservation management interventions.

Stress is a response to internal or external factors (stressors) that cause a change in the physiological condition of an animal (von Holst, 1998; Moberg and Mench 2000; Möstl and Palme, 2002; Clinchy, Sheriff and Zanette 2013; McFarland *et al.*, 2013). When a stressor is perceived by an individual, the hypothalamic-pituitary-adrenal (HPA) axis is activated, releasing glucocorticoids (cortisol and/or corticosterone) into the bloodstream (Koren *et al.*, 2012; Botía *et al.*, 2023) to enable a return to allostasis (Sapolsky *et al.*, 2000). Glucocorticoids are metabolized in the liver and subsequently excreted as metabolites in urine and faeces (Palme, 2019).

Usually, stress responses are brief; however, if the animal is exposed to stressors for a prolonged period, faecal glucocorticoid metabolites (FGMs) remain elevated, indicating chronic stress (Shave *et al.*, 2019; Fokidis *et al.*, 2023). Chronic stress can be detrimental to welfare, impairing immune system function (Anjos *et al.*, 2017), increasing mortality rates (Wey *et al.*, 2015; Narayan, 2019; Young *et al.*, 2019), and reducing fitness (Moberg and Moberg, 1985; Von Holst, 1998; Oliveira *et al.*, 2024). Chronic stress responses have been detected in mammals with physical injury (Wolf *et al.*, 2018), when infected by parasites (Seguel *et al.*, 2019; Meza-Montes *et al.*, 2023), when encountering sub-optimal resource availability (Bragg *et al.*, 2024), or when experiencing reduced habitat quality (Oliveira *et al.*, 2024).

FGMs provide a non-invasive tool to measure adrenocortical activity and stress (Palme, 2019). However, selecting the most appropriate assay for measuring FGMs in each species is essential because species differ markedly in their stress responses, and either cortisol or corticosterone (Koren *et al.*, 2012) will be the primary stress hormone for a particular species. In addition to species differences, there can also be significant differences in metabolites between males and females (Millspaugh and Washburn, 2004; Goymann, 2005; Palme, 2005, 2019). Measurement of FGMs is most frequently performed with enzyme immunoassays (EIAs). There are many different EIAs available that have been widely tested on mammalian species (Palme, 2019, 2026). Most importantly, any assay used to detect glucocorticoid metabolites in faeces needs to be both suitably sensitive to changes in stress response, and relevant—i.e. it reacts to FGMs produced in response to the stressor and can measure small changes in FGMs (Millspaugh and Washburn, 2004; Touma and Palme, 2005; Edwards *et al.*, 2025). Therefore, to preselect an assay while saving costs, a preliminary validation experiment is performed. Once this has been completed, the next step is to carry out a physiological or biological validation to show that the selected assay is effective for the given species (Goymann, 2005; Touma and Palme, 2005).

Physiological validation can be done directly with an adrenocorticotropic hormone (ACTH) challenge (Bashaw *et al.*, 2016), which requires ACTH to be injected into the subject, and the subsequent levels of glucocorticoid metabolites are measured along with the delay time between challenge and deposition. The ACTH challenge test is an invasive procedure requiring a veterinarian, and the process itself is stressful (Sheriff *et al.*, 2011; Palme, 2019). The alternative is biological validation, where naturally deposited faecal samples are collected before and after a stressor over a longer period (Touma and Palme, 2005; Palme, 2019), with FGMs acting as a biological proxy (Palme, 2012; Edwards *et al.*, 2025).

A method is required to enable conservation managers to pursue future research avenues on hedgehogs that will identify the environmental and anthropogenic factors driving changes in populations (Dantzer *et al.*, 2014; Hing *et al.*, 2016), and FGMs offer a non-invasive way to monitor population welfare (Millspaugh and Washburn, 2004; Dantzer *et al.*, 2014; Souza *et al.*, 2026). For those working in hedgehog conservation, FGM analysis may provide a valuable tool for understanding stressors. However, to date, no method has been validated to measure FGMs in hedgehogs.

This study aimed to identify a suitable assay to measure FGMs in hedgehogs by performing a preliminary validation of three potential assays. A biological validation of the most sensitive assay was carried out by comparing FGMs in hedgehog samples collected the day after admission to a rehabilitation centre, Day 1 (hereafter, ‘d1’), and in samples from the same individuals collected 7 days after admission (hereafter, ‘d7’) to demonstrate the assay’s ability to detect changes in hedgehog FGMs over time, and after a stress event. We predicted that FGM levels in individuals would decrease after receiving treatment. The assay was then used in two applications to demonstrate its utility for measuring stress responses. First, FGMs from physiologically compromised rehabilitating animals were compared between those that survived, and those that died, or were euthanized, to determine a relationship between FGMs and mortality rates. We predicted FGMs would be relatively lower in those that survived rehabilitation. A further comparison evaluated FGMs between wild hedgehogs parasitised with *Capillaria syn. Eucleus* spp*.* (hereafter, *Capillaria)*, and uninfected wild hedgehogs, with the prediction that FGMs would be higher in those with parasite presence. The results of this study are important because they identify a suitable assay for measuring stress in hedgehogs, which can be applied across various conservation contexts, thereby aiding our understanding of hedgehog population stressors.

## Materials and methods

### Ethics statement

This study was conducted between May 2022 and September 2024 with ethical approval from Nottingham Trent University (ARE212226). Wild hedgehogs were captured during spotlight surveys under licence from Natural England, held in a suitable container at the site of capture for up to four hours until they defecated, and then released.

### Sample collection and processing

The majority of faecal samples were obtained from hedgehogs admitted to Wild Hogs Hedgehog Rescue, rehabilitation centre in Gloucester, UK. A summary of the rehabilitation centre standards and admissions process is provided in the supplementary materials. Admitted animals were physiologically compromised by injury, disease, or infection, and the admission process included several stressors known to trigger a stress response in other mammals, namely capture, transport, and veterinary examination (Kenagy and Place, 2000; Palme *et al.*, 2000; Young *et al.*, 2004; Grandin and Shivley, 2015; Seguel *et al.*, 2019; Shave *et al.*, 2019; Eguizábal *et al.*, 2021). Therefore, hedgehogs were assumed to have elevated stress levels on admission.

Naturally deposited faecal samples were collected from rehabilitating hedgehogs during daily cage inspections, and samples from d1 and d7 were used to compare FGM levels after a standard period of recovery and treatment. All faeces were stored in individual sample tubes and frozen immediately at −20°C. The reported gut transit time for hedgehogs is 12–16 hours (Clemens, 1978). Samples were collected each morning, with a maximum interval of 28 hours, reflecting stress levels during the two previous gut transit periods.

Samples were also collected from 10 wild hedgehogs captured in Gloucester, UK, under licence from Natural England (2023-65 115-SCI-SCI). Hedgehogs were found during nighttime spotlight surveys and held in a large plastic container (dimensions = 62 × 38.5 × 35 cm) at the location where they were found. The container contained cat food and bedding material (pieces of dry newspaper), and captured individuals were held until they defecated, or for up to 4 hours, depending on which occurred first. Hedgehogs were checked hourly, and once they had defecated, they were weighed (to 0.01 kg), sexed, and released at the site of capture. Faecal samples were stored in a cool box during fieldwork, then frozen at −20°C within 6 hours of initial collection.

Faecal samples were defrosted at room temperature, homogenized, and steroid extraction was carried out as described by Palme *et al.* (2013). In brief, 0.5 g of wet faeces was extracted with 5 ml of 80% methanol. The sample was then vortexed for 30 minutes and centrifuged at 2500 × g for 15 minutes. An aliquot of the supernatant (0.5 ml) was pipetted into centrifuge tubes and evaporated in a water bath at 60°C before being sent to the University of Veterinary Medicine in Vienna for analysis (Import permit E2024-0196). After redissolving the extracts in 80% methanol, a 1:10 (1 + 9) dilution with assay buffer was carried out to avoid the influence of high-concentration methanol, and then 10 μl was transferred into the EIA (or up to 50 μl if needed). The first step with the three EIAs revealed that all samples lay well within the nearly linear range of the standard curve (80% to 20% binding; only a few high-concentration samples required further dilution).

### Preliminary assay validation and faecal smears

Processed faecal samples, consisting of 68 samples from 10 rehabilitating hedgehogs (six adult males, two adult females, and two juvenile females), were divided and screened for FGMs using three different group-specific EIAs. These three EIAs were chosen because they have been successfully applied to a range of mammalian species (Palme, 2019, 2026). Assays used were an 11β-hydroxyetiocholanolone EIA (hereafter, ‘69a’), an 11-oxoetiocholanolone EIA (hereafter, ‘72T’), and a 5α-pregnane-3β,11β,21-triol-20-one EIA (hereafter, ‘37e’). Assay characteristics, including cross-reactivities with different FGMs, are reported elsewhere (Möstl and Palme, 2002; Touma *et al.*, 2003; Frigerio *et al.*, 2004).

Faecal samples from wild hedgehogs were processed as described above, and the remainder of sample was refrozen. These were later defrosted at room temperature and processed following South and Haynes (2018) to determine the presence of *Capillaria* parasites. Briefly, a cotton bud was used to smear faeces onto a microscope slide, a drop of saline solution was added, and a cover slip was placed on the slide. Slides were examined visually at 10× and 40× magnification for the presence of *Capillaria*. No other endoparasites were identified in the smear.

### Biological validation

Because assay 69a yielded the highest baseline/peak ratio (i.e. greatest sensitivity) and the greatest abundance of metabolites, all subsequent analyses were conducted using this assay. In addition, a parallelism test (serial dilutions of two high-concentration samples) was performed with this EIA.

Paired samples from 18 rehabilitating hedgehogs (10 adult males, three juvenile males, four adult females, and one juvenile female) were collected the day after admission (d1) and again one week after admission (d7). Samples were compared to determine whether FGM levels had changed, indicating that the EIA could detect responses to HPA activity.

### Assay applications

The first application of assay 69a compared FGMs of hedgehogs that survived, or died, or were euthanized during rehabilitation, to assess whether the assay could detect FGMs associated with increased mortality. Faecal samples from 71 rehabilitating hedgehogs (36 adult males, seven juvenile males, 22 adult females, and six juvenile females) were collected on d1, and outcomes were recorded.

Assay 69a was then used to compare samples from 10 wild hedgehogs, five infected with the parasite *Capillaria* and five uninfected*.* Rehabilitated hedgehogs had coexisting conditions that may have affected FGM concentrations, so their samples were not used in the parasite analysis.

### Data analysis

Data analyses were carried out in R (RStudio v 2024.090 + 375, Cranberry Hibiscus release) (R Core Team, 2024). The package *dplyr* (Wickham *et al.*, 2023) was used to produce descriptive statistics, and the *outliers* package (Komsta, 2022) was used to carry out Grubb’s test for outliers in each dataset. The package *car* (Fox and Weisberg, 2019) was used to test for homogeneity. All graphs in this analysis were produced using the package *ggplot2* (Wilkinson, 2011). Odds ratios were calculated using the package *broom* (Robinson, Couch, and Hvitfeldt, 2026).

Preliminary assay validation datasets were not normally distributed or homogeneous, so a Kruskal–Wallis test (Knapp, 2017) was performed to assess differences between assays.

A paired Wilcoxon test (McCullagh, 2018) was used for the biological validation to determine whether differences in FGMs between d1 and d7 were significant.

FGM concentrations were also analysed by rehabilitation survival outcome (i.e. died/euthanized vs survived), and a Mann–Whitney U test (Costescu, 2008) was performed to assess whether FGM measurements differed between those that survived and were released, and those that died or were euthanized. FGM concentrations and outcomes were then modelled to establish whether increasing FGM concentrations were related to a greater probability of death or euthanasia during rehabilitation. A binomial Gaussian linear model (Smith *et al.*, 2020) was used to create a logistic regression model of FGM concentrations and survival outcomes. The logistic regression model was trained using 70% of the dataset and tested on the remaining 30%. A McFadden pseudo *R*^2^ value was calculated to assess the model (Poston *et al.*, 2023).

To assess the model’s performance in predicting outcomes from FGM concentrations, a receiver operating characteristic curve was calculated (see Supplementary Material, Supplementary Figure S1). This modelled the number of true positives (sensitivity) against the number of false positives (specificity) to ensure the model performed better than chance alone. The area under the curve value of the model (Hosmer *et al.*, 2013) was calculated, and values range from 0 to 1. Values above 0.5 indicated that the model performed better than chance alone (model value 0.73). Odds ratios were used to calculate survival rates at different FGM levels.

For comparisons of parasite-infected animals, data were normally distributed, and an unpaired *t*-test was used to determine whether hedgehogs with or without *Capillaria* had significantly different FGM levels.

## Results

### Preliminary assay validation

Serial samples from 10 individual hedgehogs were tested using three assays to determine the range of FGMs detected by each assay (Table 1). All assays tested were sufficiently sensitive to detect changes in hedgehog FGMs. However, a comparison of the assays showed that assay 69a yielded a significantly greater range of FGM levels than the other assays (Kruskal–Wallis test, χ2 = 51.3, df = 2, *P* < 0.001) and detected the greatest abundance of metabolites, with overall percentages of peak increase being highest (with a few exceptions).

**Table 1:** Faecal samples were collected daily from 10 Western European hedgehogs over 51 days during the rehabilitation process

		**Range of FGM concentrations [peak increase in %]**		
**Hedgehog ID**	**Number of samples**	**69a (ng/g) [sensitivity]**	**72T (ng/g) [sensitivity]**	**37e (ng/g) [sensitivity]**
Male 1	9	58–425 [731%]	53–192 [358%]	15–118 [794%]
Male 2	8	80–601 [748%]	58–480 [834%]	20–130 [636%]
Male 3	5	196–620 [316%]	107–337 [316%]	41–171 [423%]
Male 4	12	36–774 [2156%]	34–604 [1804%]	14–286 [2062%]
Female 1	7	180–1423 [793%]	124–940 [761%]	86–456 [532%]
Female 2	9	64–387 [604%]	60–306 [512%]	38–136 [362%]
Female 3	7	101–822 [811%]	80–585 [727%]	35–501 [1421%]
Female 4	7	345–1219 [354%]	238–683 [287%]	91–308 [339%]
Female 5	3	393–985 [251%]	298–503 [169%]	117–150 [128%]
Female 6	1	763	879	365
Overall	68	36–1423	34–940	14–501

Samples were analysed for faecal glucocorticoid metabolites (FGMs) using three enzyme immunoassays (EIAs): 11β-hydroxyetiocholanolone EIA (hereafter, ‘69a’), the 11-oxoetiocholanolone EIA (hereafter ‘72T’), and the 5α-pregnane-3β,11β,21-triol-20-one EIA (hereafter, ‘37e’). The ranges of responses, together with peak increase in percentage, are presented for each hedgehog.

**Figure 1:**
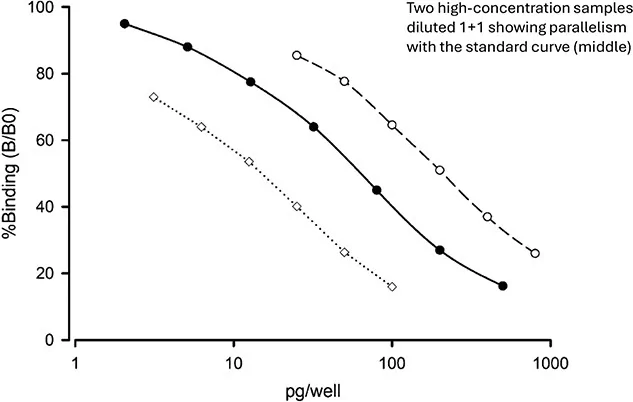
Two high-concentration samples diluted 1 + 1 showing parallelism.

The analytical sensitivity of assay 69a was 2.1 ng/g. Inter-assay coefficients of variation of a low and high pool sample (the variation of the sample between plates) were 8.4% and 11.0%, respectively. Serial dilutions of extracts containing high FGM concentrations were performed and yielded curves parallel to the standard curve (Fig. 1).

### Biological validation

**Figure 2:**
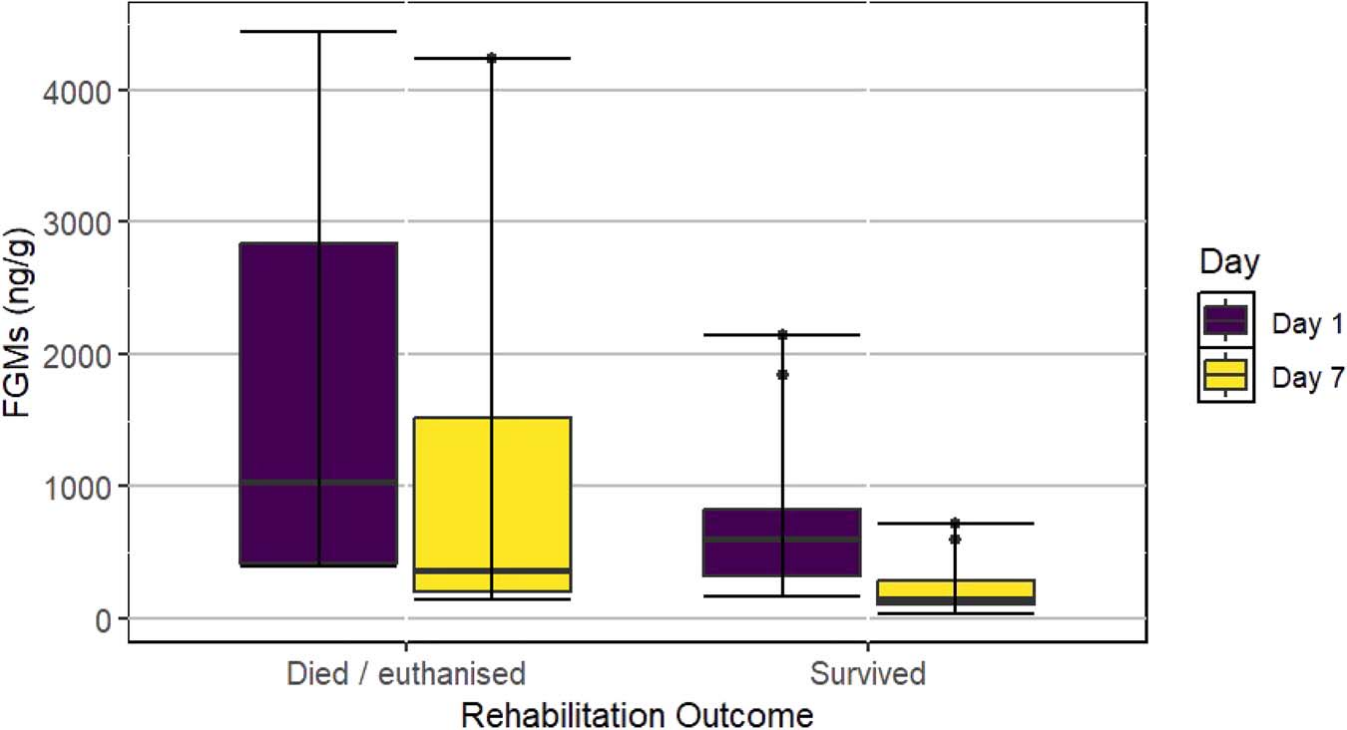
Boxplot of faecal glucocorticoid metabolite concentrations (FGM) measured using the 11β-hydroxyetiocholanolone enzyme immunoassay (69a EIA). Faecal samples were collected at a UK hedgehog rehabilitation centre from 18 Western European hedgehogs (five died or were euthanized, 13 survived) on day 1 (d1) and day 7 (d7) after admission. Boxplot represents the minimum, median (line), and maximum values, with the range represented by lines at the ends of the whiskers. The box represents 50% of the values, and the dots show any outliers plotted on the *y*-axis (FGM concentrations). Differences between d1 and d7 were significantly lower on d7 for surviving hedgehogs (Wilcoxon test, W = 78, *n* = 13, *P* = 0.003), but not for those that died or were euthanized (Wilcoxon test, W = 11, *n* = 5, *P* = 0.4). FGM concentrations are reported in nanograms per gram wet faeces.

For the biological validation, FGMs were highest on d1 (mean 743 ng/g, SD = 602.7) for all rehabilitating hedgehogs, and reduced by d7 (mean 224 ng/g, SD = 207.5). This difference was significant for hedgehogs that survived rehabilitation only (Wilcoxon test, W = 78, *n* = 13, *P* = 0.003) (Fig. 2).

### Assay applications

FGMs for rehabilitating hedgehogs by outcome are shown in Table 2, along with a comparison of the FGMs found in hedgehogs captured in the wild. The highest FGM concentration in a hedgehog that survived rehabilitation was 5332 ng/g on the day of admission. This individual was wounded by a dog but went on to make a full recovery. All other hedgehogs with FGM concentrations over 5332 ng/g on the day of admission were euthanized on welfare grounds, or died.

**Table 2:** A summary of faecal glucocorticoid metabolite (FGM) concentrations reported in nanograms per gram of wet faeces for 71 Western European hedgehogs from naturally deposited samples collected from the day after admission (Day 1) to rehabilitation in the UK and tested for FGMs using enzyme immunoassay 11β-hydroxyetiocholanolone assay (69a EIA). Median given in nanograms per gram of wet faeces. Highlighted section shows the range of FGMs for all rehabilitating hedgehogs and the median in nanograms per gram of wet faeces.

**Rehabilitation outcome/wild**	**Number of samples**	**Range of FGM concentrations (ng/g)**	**Median (ng/g)**
Rehabilitating—died	15	747–34 672	2968
Rehabilitating—euthanized	6	388–27 771	3000
Rehabilitating—survived	50	56–5332	708
**All rehabilitating hedgehogs**	**71**	**56–34 672**	**888**
Wild hedgehogs	10	117–1284	378

10 wild hedgehogs were captured during spotlight surveys and held in a suitable containers for up to 4 hours until defecation occurred. The number of samples is shown, and FGM ranges are reported in nanograms per gram wet faeces. The minimum, maximum, and median FGM concentrations are presented in the table.

FGM levels from wild hedgehogs had the lowest median concentrations, and the lowest range of all hedgehog cohorts in this study (Table 2).

The two hedgehogs with low FGMs on admission (388 ng/g and 412 ng/g) that were subsequently euthanized both developed complications later during the rehabilitation process (one collapsed after d13, the other had untreatable alopecia). All other hedgehogs that were euthanized had FGMs over 3000 ng/g on the day of admission.

**Figure 3:**
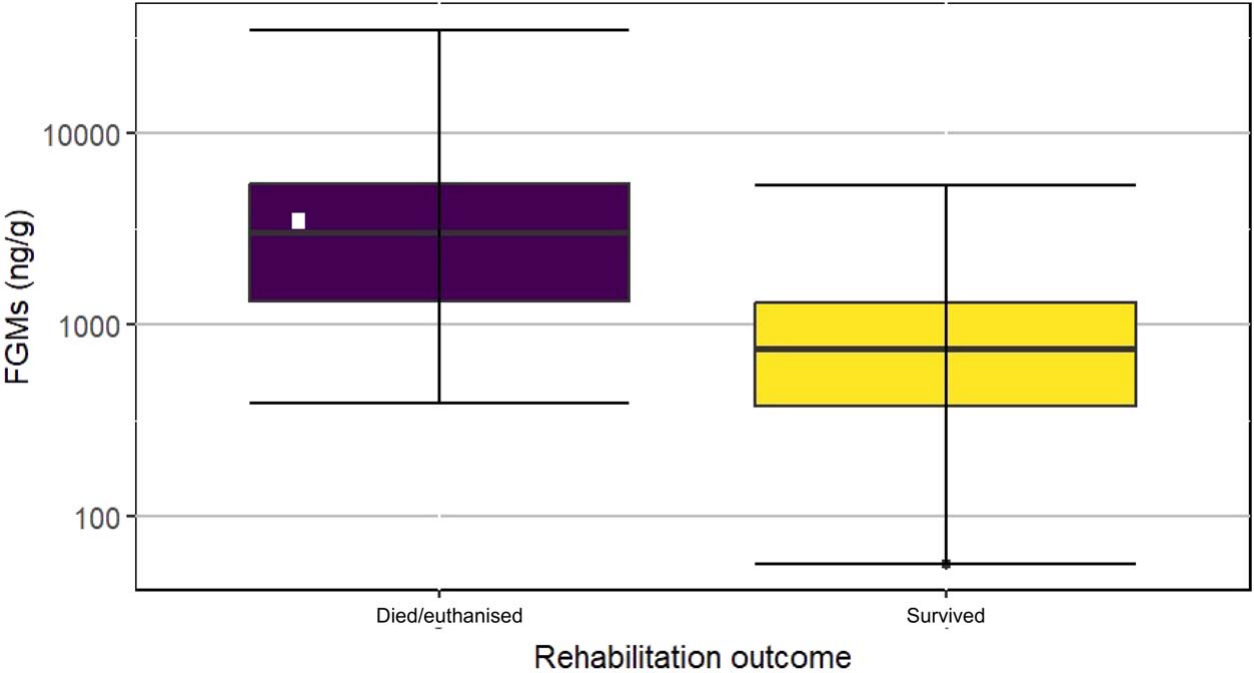
Boxplot of faecal glucocorticoid metabolite (FGM) concentrations measured using the 11β-hydroxyetiocholanolone enzyme immunoassay (69a EIA). Faecal samples from 71 Western European hedgehogs admitted to rehabilitation were collected the day after admission and analysed according to outcome. Rehabilitation admissions that survived had significantly lower FGM concentrations on the first day of admission than those that died or were euthanized (Mann–Whitney U test, W = 873, *n* = 71, *P* < 0.001). A boxplot represents the minimum, median (midline), and maximum values, with the range indicated by lines at the ends of the whiskers. The box represents 50% of the values, and the dots show any outliers plotted on the *x*-axis (rehabilitation outcome) and *y*-axis (FGM concentrations), on a logarithmic scale (base 10), and reported in nanograms per gram (ng/g) wet faeces.

FGM levels on admission were significantly lower for hedgehogs that survived compared to those that died, or were euthanized (Mann–Whitney U test, W = 873, *n* = 71, *P* < 0.001; Fig. 3).

**Figure 4:**
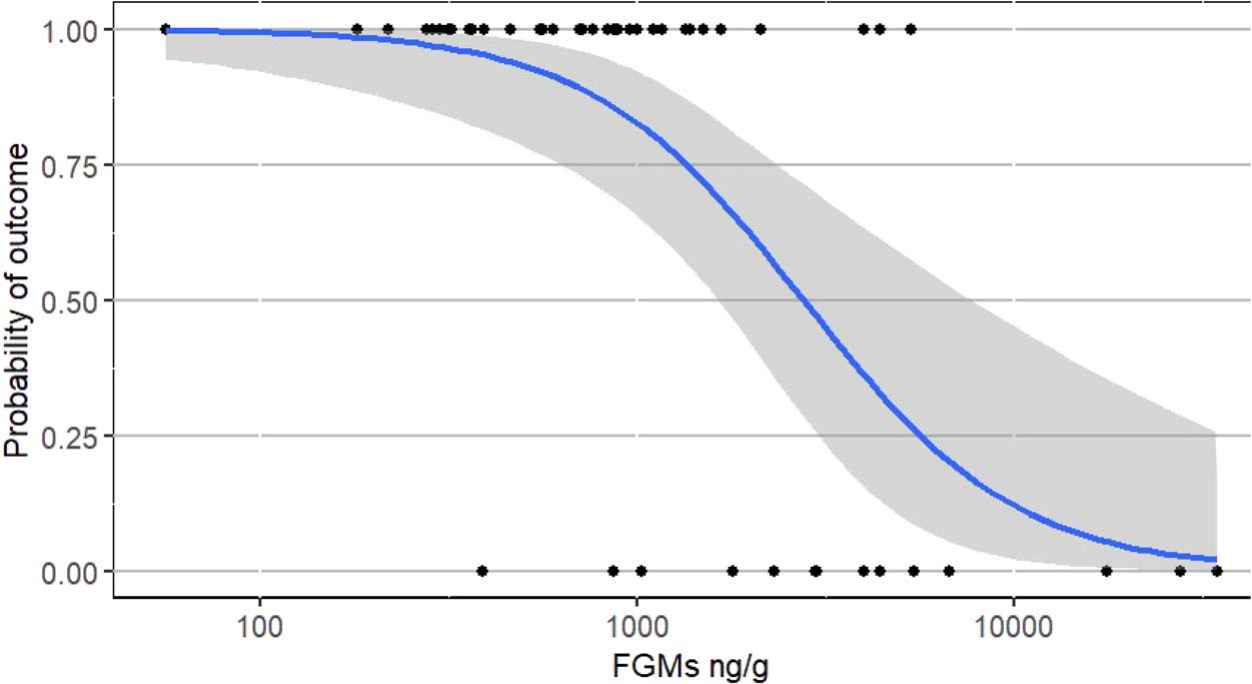
Faecal glucocorticoid metabolite (FGM) concentrations measured using the 11β-hydroxyetiocholanolone enzyme immunoassay (69a EIA) and the probability of outcomes 1 = survived, 0 = died or were euthanized. The shaded area represents a 95% confidence interval. Faecal samples from 71 Western European hedgehogs admitted to rehabilitation were collected the day after admission and modelled using a binomial Gaussian linear model (GLM; logistic regression) against outcomes (0 = died or were euthanized, 1 = survived). FGM concentrations are plotted on a logarithmic scale (base 10) and reported in nanograms per gram (ng/g). The odds of dying were calculated as −0.0007 (SE = 0.0002). Overall model fit was 33% (McFadden Pseudo *R*^2^ = 0.33).

The odds of an individual hedgehog dying after admission were calculated as −0.0007 (Standard Error = 0.0002) as measured on d1. Overall model fit was 33% (McFadden pseudo *R*^2^ = 0.33; Fig. 4).

The model was used to predict the probability of survival for hedgehogs with different FGM levels measured in faecal samples on the day of admission to rehabilitation (Table 3).

**Table 3:** The probability of a hedgehog surviving rehabilitation at a UK rehabilitation centre, with different levels of FGMs in faecal samples

**FGMs (ng/g)**	**Probability of survival**
1000	82.8%
3000	52.4%
5000	20.1%
7500	3.8%
10 000	0.6%
20 000	0.0%

Faecal glucocorticoid metabolites (FGMs) in samples were measured with the 11β-hydroxyetiocholanolone enzyme immunoassay (69a EIA). Faecal samples from 71 Western European hedgehogs admitted to rehabilitation were collected the day after admission, and analysed according to outcome.

**Figure 5:**
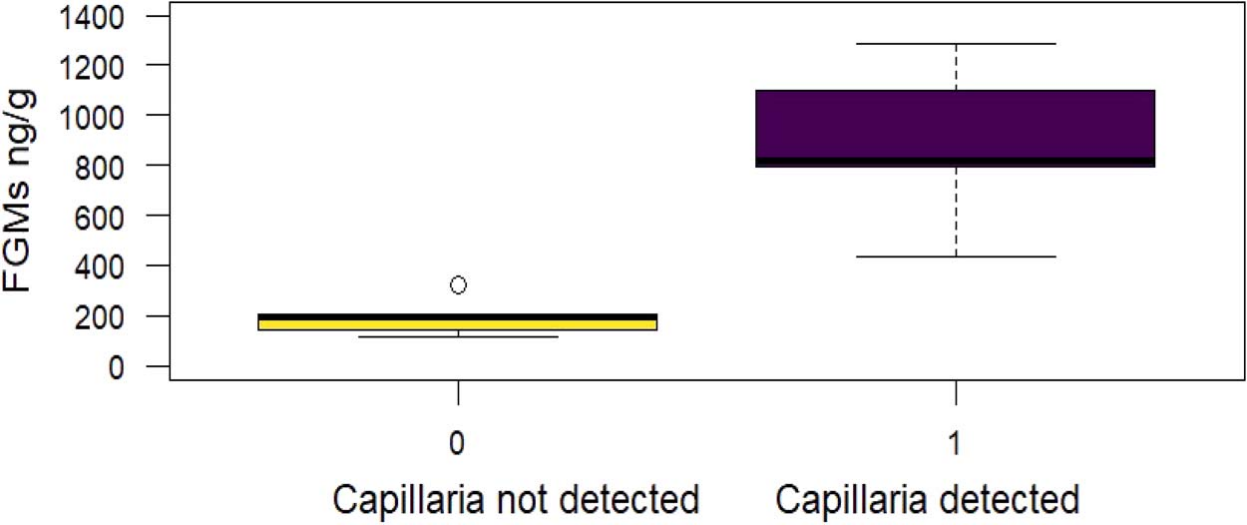
Boxplot of faecal glucocorticoid metabolite (FGM) concentrations tested using the 11β-hydroxyetiocholanolone enzyme immunoassay (69a EIA). Faecal samples were collected from 10 wild hedgehogs that were captured during spotlight surveys and held in suitable containers for up to 4 hours until they defecated. Faecal smears were used to identify whether samples contained the parasitic nematode *Capillaria*. The boxplot shows the minimum, median (midline), and maximum FGM values, with the range indicated by lines at the ends of the whiskers. The box represents 50% of the FGM values, and the dots mark any outliers plotted on the *x*-axis (*Capillaria* present or absent) and *y*-axis (FGM concentrations), reported in nanograms per gram (ng/g) wet faeces.

The 69a assay was also applied to faecal samples from 10 wild hedgehogs with and without the presence of *Capillaria* (five present, five absent). No other endoparasites were identified in the smear. There were significantly higher FGM concentrations in the samples from individuals where *Capillaria* was present compared to the samples where *Capillaria* was absent (unpaired *t*-test, t_4.5_ = −4.6, *P* = 0.007; Fig. 5).

Of the infected hedgehogs, two were admitted to rehabilitation for further treatment. Of the three hedgehogs with *Capillaria* that were not admitted to rehabilitation, each was assessed at the time of capture, found to have no injuries or other clinical symptoms, and weighed around 800 g, and thus remained in the wild.

## Discussion

This study successfully identified a suitably sensitive assay that can be used to measure FGM concentrations in hedgehogs exposed to different stressor contexts. While the preliminary validation showed all three assays demonstrated variation in FGMs, the 11ß-hydroxyetiocholanolone EIA (assay 69a) was the most sensitive to changes in FGM concentrations, with 37e and 72T being less sensitive and less effective at identifying metabolites. This underlines the necessity for checking several EIAs when performing validation experiments to find the most sensitive one (Palme, 2019).

The biological validation of assay 69a demonstrated that the assay was effective in detecting changes in individual FGM levels through time, evidencing its utility as a proxy for measuring stress in this species. As predicted, individuals that survived rehabilitation showed relatively higher FGM levels upon admission compared to samples from the same individuals after a week of treatment. It is likely that the combined provision of care, food, water, and veterinary treatment, reduced stress levels by mitigating the cause of stress, and this was reflected in the lower FGM levels after seven days. Furthermore, FGM levels in the surviving individuals had reduced to a mean of 224 ng/g (SD = 207.5) by d7, which was similar to the observed range (range 117–322 ng/g) for wild uninfected hedgehogs reported here. By contrast, those that died or were euthanized, continued to show relatively high FGM concentrations after seven days of care, presumably because the cause of stress they were experiencing upon admission was chronic and beyond the point where treatment could be effective. The comparatively high FGM levels in those that died further evidences the link between individuals struggling to survive and physiological stress responses in this species.

Wild animals experiencing chronic stress will have compromised immune system function (Anjos *et al.*, 2017), and experience higher risks of mortality (Wey *et al.*, 2015; Narayan, 2019; Young *et al.*, 2019). As predicted, we also found a relationship between elevated FGMs on the day of admission and the likelihood of an individual surviving the rehabilitation process. None of the surviving hedgehogs had FGM concentrations above 5332 ng/g on admission, while many of those that died or were euthanized had FGM concentrations below this, which is likely linked to individual ailment contexts. We propose that using assay 69a to measure relative stress levels of individuals admitted to rehabilitation, and linking this to the probable cause of admission, could be used to better understand and evidence euthanasia decision-making by veterinarians. For example, hedgehogs are admitted to rehabilitation for a number of causes, such as physical trauma from a dog bite, poor body condition, high parasite burdens, or exhibiting abnormal behaviours such as being out during the day, and often combinations of these causes are identified (Martínez *et al.*, 2014; Burroughes *et al.*, 2021; Mullineaux and Pawson, 2023; Vezyrakis *et al.*, 2023; White *et al.*, 2024). Further research to model the stress levels associated with causes of admission and the likelihood of survival would improve decisions on euthanasia, reduce prolonged suffering in individuals with low chances of survival, and improve the welfare of rehabilitated hedgehogs generally.

We also demonstrated the application of FGM measurements using assay 69a in response to a parasite-induced stressor. Parasite burdens can compromise individual health, elevating stress responses, and in high-intensity situations can result in mortality (Stoks, 2001; McCauley *et al.*, 2011; MacLeod *et al.*, 2018). As predicted, FGM concentrations were higher in wild hedgehogs infected with the endoparasite *Capillaria* compared to uninfected animals. Hedgehogs are well-known hosts for a range of parasites, from ectoparasites such as ticks (*Ixodes* spp.) and fleas (*Archaeopsylla erinaceid*), to endoparasites such as lungworm (*Crenosoma striatum*) and *Capillaria* (Boag and Fowler, 1988; Gaglio *et al.*, 2010; Haigh *et al.*, 2014; Pfäffle, 2015). The influence of these parasites and the resulting diseases on hedgehog population dynamics, and their contribution to population declines is limited (PfÄFfle *et al.*, 2009; Budischak *et al.*, 2012). Therefore, understanding how varying parasite burdens influence hedgehog welfare can be informed by studies using assay 69a to measure FGM levels in hedgehogs with different parasites and burdens. Comparing and contrasting FGM levels in hedgehogs with different pathologies would help to identify which are the greatest threat to individual fitness, and ultimately how this may translate into population-level effects.

The only previous hedgehog FGM concentration study (Rasmussen *et al.*, 2021) used unvalidated assays with untested cross-species reactivity that are typically applied to measure human stress in cell culture supernates, serum, plasma, saliva, and urine (“Corticosterone ELISA”, REFERENCE EIA 4164; DRG Instruments GmbH, Germany, and “Parameter cortisol assay”, KGE008, R & D Systems Parameter Cortisol). These assays were used to measure saliva corticosterone, faecal corticosterone metabolites levels, and faecal cortisol levels in wild and rehabilitated hedgehogs (Rasmussen *et al.*, 2021). The study detected higher levels of faecal corticosterone metabolites and saliva corticosterone in rehabilitated animals than in wild hedgehogs. However, no significant link between survival and FGM concentrations was identified in the study, which differs from the findings here. Assay validation is an essential step in non-invasive FGM studies (Touma and Palme, 2005; Palme, 2019). Without validation, results may fail to detect meaningful changes and may even lead to inaccurate findings (Britnell *et al.*, 2024), and the choice of assay selection may explain the differences between this study and Rasmussen *et al.* (2021). We therefore recommended that assay 69a be used to measure FGMs as a proxy for stress in future hedgehog studies.

A potential confounding factor in our study was the possibility that rehabilitated individuals were experiencing additional stress associated with captivity, as has been demonstrated in other wild species (Fischer and Romero, 2019). However, for the rehabilitated surviving individuals, FGM levels after seven days of treatment were similar to those observed in the wild individuals, suggesting no evidence of captivity-induced stress in this cohort. The time hedgehogs remain in rehabilitation varies (Morris, 1997; Molony *et al.*, 2006), but it can represent a significant proportion of an individual’s life if it is retained in care over winter, for example (Yarnell *et al.*, 2019). Other studies have shown hedgehogs can survive long periods in rehabilitation and be successfully released back into the wild (Morris, 1997; Molony *et al.*, 2006), and there is no evidence that prolonged rehabilitation is detrimental to hedgehog welfare. Factors such as the conditions in which hedgehogs are kept, and the treatments given, may influence the levels of captivity-induced stress experienced (Rothschild *et al.*, 2008; Green *et al.*, 2025). Whether this leads to chronic stress and impacts longer-term fitness and survival within rehabilitation, or once individuals are released, remains to be tested. However, we advocate returning rehabilitated hedgehogs to the wild as soon as they are deemed healthy by a veterinarian, to ensure that any potential for captivity-induced stress is minimized.

FGM levels remained high in hedgehogs that did not survive rehabilitation, and we were unable to determine whether these high levels were due solely to the cause of admission, or whether their physiological condition was further compromised by stressors associated with being in captivity. Further case studies using assay 69a to quantify relative stress levels associated with various hedgehog admission causes, and potential additional stressors linked to care, would also help inform hedgehog welfare management practises in rehabilitation. It is estimated that more than 40 000 hedgehogs are admitted to rehabilitation facilities every year in Britain, with about half returned to the wild, potentially representing an important factor in the population dynamics of Britain’s hedgehogs (Bearman-Brown and Baker, 2022). Ultimately, the more hedgehogs successfully rehabilitated, the greater the likely benefits to local populations in terms of reducing mortality rates and maintaining population viability. Further physiological research could help identify the conditions and treatments that reduce stress in hedgehogs and increase rehabilitation success.

Sex and age difference can influence FGMs within species (Millspaugh and Washburn, 2004; Goymann, 2005; Touma and Palme, 2005). Unfortunately, we were unable to factor in age and sex due to individual variation among physiologically compromised animals, which could not be controlled for in this study. We recommend that future hedgehog studies confirm any differences by sex and age to improve the typical baseline levels of FGMs reported here. Further exploration of how FGM levels vary seasonally in wild hedgehogs would also help identify periods of potential stress experienced throughout the year, such as droughts when macroinvertebrates and water availability may impact the physiological condition, post-reproduction in females, or periods following hibernation when fat reserves are lowest and when hedgehogs have been shown to experience higher mortality rates (Yarnell *et al.*, 2019; Bearman-Brown *et al.*, 2020). Using FGMs as a tool to measure stress experienced by individuals in this way could provide insight into identifying periods of high risk to hedgehog survival, and lead to conservation management plans implemented to mitigate these risks.

In conclusion, this study is the first to validate methods to measure FGMs in Western European hedgehogs and to recommend a suitably sensitive assay (69a EIA) for detecting changes in HPA activity following exposure to a range of stressors. By using assay 69a to measure FGMs in hedgehogs, we were able to demonstrate relationships between stressor events associated with increased mortality, parasite infection, and FGMs. The way is now open to use FGM measurements to better understand environmental and anthropogenic stressors that may be impacting individual fitness and wider population dynamics in declining hedgehogs. We therefore recommend the use of 69a as a non-invasive sampling tool suitable for assessing hedgehog stress both in captivity and in the wild, and to test stressors associated with current and future management contexts.

## Supplementary Material

Web_Material_coag063

## Data Availability

The data underlying this article are available at Zenodo repository: https://doi.org/10.5281/zenodo.16792090.

## References

[ref1] Anjos L, Cardoso JCR, Mateus A, Power D (2017) Chronic stress impairs the local immune response during cutaneous repair in Gilthead Sea bream (*Sparus aurata*, L.). Mol Immunol 87: 267–283. 10.1016/j.molimm.2017.04.008. 28521279

[ref2] Bashaw MJ, Sicks F, Palme R, Schwarzenberger F, Tordiffe ASW, Ganswindt A (2016) Non-invasive assessment of adrenocortical activity as a measure of stress in giraffe *(Giraffa camelopardalis)*. BMC Vet Res 12: 235. 10.1186/s12917-016-0864-8. 27756312 PMC5070010

[ref3] Bearman-Brown L, Baker P (2022) An estimate of the scale and composition of the hedgehog *(Erinaceus europeaus)* rehabilitation community in Britain and the Channel Islands. Animals (Basel) 12: 3139. 10.3390/ani12223139. 36428367 PMC9686839

[ref4] Bearman-Brown LE, Baker PJ, Scott D, Uzal A, Evans L, Yarnell RW (2020) Over-winter survival and nest site selection of the west-European hedgehog (*Erinaceus europaeus*) in arable dominated landscapes. Animals (Basel) 10: 1422–1449. 10.3390/ani10091449. 32825054 PMC7552789

[ref5] Berger A, Barthel LMF, Rast W, Hofer H, Gras P (2020) Urban hedgehog behavioural responses to temporary habitat disturbance versus permanent fragmentation. Animals (Basel) 10: 2109–2119. 10.3390/ani10112109. 33203020 PMC7697271

[ref6] Berger A, Lozano B, Barthel LMF, Schubert N (2020) Moving in the dark—evidence for an influence of artificial light at night on the movement behaviour of European hedgehogs (*Erinaceus europaeus*). Animals 10: 1306. 10.3390/ani10081306. 32751525 PMC7459628

[ref7] Boag B, Fowler PA (1988) The prevalence of helminth parasites from the hedgehog *Erinaceus europaeus* in Great Britain. J Zool 215: 379–382. 10.1111/j.1469-7998.1988.tb04908.x.

[ref8] Botía M, Escribano D, Martínez-Subiela S, Tvarijonaviciute A, Tecles F, López-Arjona M, Cerón JJ (2023) Different types of glucocorticoids to evaluate stress and welfare in animals and humans: general concepts and examples of combined use. Metabolites 13: 106. 10.3390/metabo13010106. 36677031 PMC9865266

[ref9] Bragg M, Muletz-Wolz CR, Songsasen N, Freeman EW (2024) Kibble diet is associated with higher faecal glucocorticoid metabolite concentrations in zoo-managed red wolves *(Canis rufus)*. Conserv Physiol 12: coae008. 10.1093/conphys/coae008. 38414659 PMC10898788

[ref10] Britnell JA, Palme R, Kerley GIH, Jackson J, Shultz S (2024) Previous assessments of faecal glucocorticoid metabolites in cape mountain zebra *(Equus zebra zebra)* were flawed. Funct Ecol 38: 1862–1874. 10.1111/1365-2435.14621.

[ref11] Budischak SA, Jolles AE, Ezenwa VO (2012) Direct and indirect costs of co-infection in the wild: linking gastrointestinal parasite communities, host hematology, and immune function. Int J Parasitol Parasites Wildl 1: 2–12. 10.1016/j.ijppaw.2012.10.001. 24533308 PMC3904086

[ref12] Burroughes ND, Dowler J, Burroughes G (2021) Admission and survival trends in hedgehogs admitted to RSPCA wildlife rehabilitation centres. Proc Zool Soc 74: 198–204. 10.1007/s12595-021-00363-9.

[ref13] Clemens ET (1980) The digestive tract: insectivore, prosimian, and advanced primate. In Schmidt-Nielson K, Bolis L, Taylor CR (eds) Comparative Physiology: Primitive Mammals (4th International Conference on Comparative physiology held at Crans-sur-Sierre, Switzerland, 4–9 June 1978). Cambridge University Press, pp. 90–99.

[ref75] Clinchy M, Sheriff MJ, Zanette LY (2013) Predator-induced stress and the ecology of fear. Functional ecology 27: 56–65.

[ref14] Costescu MR (2008) Verifying data homogeneity. Applied program u test (Mann-Witney) for comparing two empirical distributions. *Analele Universitatii din Craiova*-*Seria stiinte economice* XXXVI: 3181–3185.

[ref15] Dantzer B, Fletcher QE, Boonstra R, Sheriff MJ (2014) Measures of physiological stress: a transparent or opaque window into the status, management and conservation of species? Conserv Physiol 2: cou023. 10.1093/conphys/cou023. 27293644 PMC4732472

[ref16] Edwards KL, Wheaton CJ, Brown JL, Dimovski AM, Fanson KV, Ganswindt A, Ganswindt SB, Hagenah N, Keeley T, Möstl E, et al. (2025) Development of an 11-oxoetiocholanolone mini-kit for the quantification of faecal glucocorticoid metabolites in various wildlife species. Conserv Physiol 13: coaf074. 10.1093/conphys/coaf074. 41142957 PMC12552035

[ref17] Eguizábal GV, Superina M, Palme R, Asencio CJ, Villarreal DP, Borrelli L, Busso JM (2021) Non-invasive assessment of the seasonal stress response to veterinary procedures and transportation of zoo-housed lesser anteater *(Tamandua tetradactyla)*. Animals (Basel) 12: 75. 10.3390/ani12010075. 35011182 PMC8744720

[ref18] Finch D, Smith B, Marshall C, Coomber F, Kubasiewicz L, Anderson M, Wright P, Mathews F (2020) Effects of artificial light at night (ALAN) on European hedgehog activity at supplementary feeding stations. Animals (Basel) 10: 768. 10.3390/ani10050768. 32354129 PMC7278375

[ref19] Fischer CP, Romero LM (2019) Chronic captivity stress in wild animals is highly species-specific. Conserv Physiol 7: coz093. 10.1093/conphys/coz093. 31824674 PMC6892464

[ref20] Fokidis HB, Brock T, Newman C, Macdonald DW, Buesching CD (2023) Assessing chronic stress in wild mammals using claw-derived cortisol: a validation using European badgers (*Meles meles*). Conserv Physiol 11: coad024. 10.1093/conphys/coad024. 37179707 PMC10171820

[ref21] Fox J, Weisberg S (2019) An {R} Companion to Applied Regression. Sage Publications, CA

[ref22] Frigerio D, Dittami J, Möstl E, Kotrschal K (2004) Excreted corticosterone metabolites co-vary with ambient temperature and air pressure in male Greylag geese (*Anser anser*). Gen Comp Endocrinol 137: 29–36. 10.1016/j.ygcen.2004.02.013. 15094333

[ref23] Gaglio G, Allen S, Bowden L, Bryant M, Morgan ER (2010) Parasites of European hedgehogs (*Erinaceus europaeus*) in Britain: epidemiological study and coprological test evaluation. Eur J Wildl Res 56: 839–844. 10.1007/s10344-010-0381-1.

[ref24] Gazzard A, Rasmussen S (2024) Erinaceus europaeus. The IUCN Red List of Threatened Species 2024: e.T29650A213411773. 10.2305/IUCN.UK.2024-2.RLTS.T29650A213411773.en.

[ref25] Gimmel A, Eulenberger U, Liesegang A (2021) Feeding the European hedgehog (*Erinaceus europaeus L.*)—risks of commercial diets for wildlife. J Anim Physiol Anim Nutr 105: 91–96. 10.1111/jpn.13561. 34247429

[ref26] Goymann W (2005) Noninvasive monitoring of hormones in bird droppings: physiological validation, sampling, extraction, sex differences, and the influence of diet on hormone metabolite levels. Ann N Y Acad Sci 1046: 35–53. 10.1196/annals.1343.005. 16055842

[ref27] Grandin T, Shivley C (2015) How farm animals react and perceive stressful situations such as handling, restraint, and transport. Animals (Basel) 5: 1233–1251. 10.3390/ani5040409. 26633523 PMC4693213

[ref28] Green J, Schmidt-Burbach J, Hartley-Backhouse L (2025) Giants in tourism: captive conditions, industry trends, and animal welfare implications for Asian elephants in tourism from 2014 to 2020. Front Ethol 4: 1532995. 10.3389/fetho.2025.1532995.

[ref29] Haigh A, O'Keeffe J, O'Riordan RM, Butler F (2014) A preliminary investigation into the endoparasite load of the European hedgehog (*Erinaceus europaeus*) in Ireland. Mammalia 78: 103–107. 10.1515/mammalia-2013-0032.

[ref30] Hing S, Narayan BEJ, Thompson A, Godfrey SS (2016) The relationship between physiological stress and wildlife disease: consequences for health and conservation. Wildl Res 43: 51–60. 10.1071/WR15183.

[ref31] Hitchcock K, Tollington S, Khwaja H, Williams LJ, Hamill K, Yarnell RW (2025) Food over features: supplementary feeding has the strongest influence on hedgehog (*Erinaceus europaeus*) occupancy in urban gardens. Urban Ecosyst 28: 225. 10.1007/s11252-025-01840-1.

[ref32] Hof AR, Bright PW (2016) Quantifying the long-term decline of the west European hedgehog in England by subsampling citizen-science datasets. Eur J Wildl Res 62: 407–413. 10.1007/s10344-016-1013-1.

[ref33] von Holst D (1998) The Concept of Stress and Its Relevance for Animal Behavior Vol 27. Elsevier Science & Technology, United States, pp. 1–131

[ref34] Hosmer DW, Lemeshow S, Sturdivant RX (2013) Applied Logistic Regression. Wiley, Hoboken.

[ref35] Kenagy GJ, Place NJ (2000) Seasonal changes in plasma glucocorticosteroids of free-living female yellow-pine chipmunks: effects of reproduction and capture and handling. Gen Comp Endocrinol 117: 189–199. 10.1006/gcen.1999.7397. 10642441

[ref36] Knapp H (2017) Kruskal-wallis test. SAGE Publications Ltd, United Kingdom.

[ref37] Komsta L (2022). Outliers: Tests for Outliers. https://CRAN.R-project.org/package=outliers.

[ref38] Koren L, Whiteside D, Fahlman Å, Ruckstuhl K, Kutz S, Checkley S, Dumond M, Wynne-Edwards K (2012) Cortisol and corticosterone independence in cortisol-dominant wildlife. Gen Comp Endocrinol 177: 113–119. 10.1016/j.ygcen.2012.02.020. 22449618

[ref39] Lowry H, Lill A, Wong BBM (2013) Behavioural responses of wildlife to urban environments. Biol Rev 88: 537–549. 10.1111/brv.12012. 23279382

[ref40] MacLeod KJ, Krebs CJ, Boonstra R, Sheriff MJ (2018) Fear and lethality in snowshoe hares: the deadly effects of non-consumptive predation risk. Oikos 127: 375–380. 10.1111/oik.04890.

[ref41] Martínez JC, Rosique AI, Royo MS (2014) Causes of admission and final dispositions of hedgehogs admitted to three wildlife rehabilitation centers in eastern Spain. Hystrix 25: 107–110.

[ref42] McCauley SJ, Rowe L, Fortin M-J (2011) The deadly effects of “nonlethal” predators. Ecology 92: 2043–2048. 10.1890/11-0455.1. 22164828

[ref43] McCleery RA (2009) Changes in fox squirrel anti-predator behaviors across the urban–rural gradient. Landsc Ecol 24: 483–493. 10.1007/s10980-009-9323-2.

[ref44] McCullagh P (2018) Tensor Methods in Statistics. Dover Publications, Berkeley.

[ref94] McFarland R, MacLarnon A, Heistermann M, Semple S (2013) Physiological stress hormone levels and mating behaviour are negatively correlated in male rhesus macaques (Macaca mulatta). Animal biology (Leiden, Netherlands) 63: 331–341.

[ref45] Meza-Montes E, Fernández-Gómez RA, Llanes-Quevedo A, Navarro-Sigüenza AG, Santiago-Alarcon D, Sosa-López JR (2023) Vocal behaviour, parasitic infection, chronic stress and body condition in rufous-naped wrens (*Campylorhynchus rufinucha*). Ibis (London, England) 165: 676–684. 10.1111/ibi.13130.

[ref46] Millspaugh JJ, Washburn BE (2004) Use of fecal glucocorticold metabolite measures in conservation biology research: considerations for application and interpretation. Gen Comp Endocrinol 138: 189–199. 10.1016/j.ygcen.2004.07.002. 15364201

[ref47] Moberg GP, Moberg GP (1985) Animal Stress. Springer, New York.

[ref93] Moberg GP, Mench JA (2000) The biology of animal stress : basic principles and implications for animal welfare / edited by GP Moberg and JA Mench. pp 44–57.

[ref48] Molony SE, Dowding CV, Baker PJ, Cuthill IC, Harris S (2006) The effect of translocation and temporary captivity on wildlife rehabilitation success: an experimental study using European hedgehogs (*Erinaceus europaeus*). Biol Conserv 130: 530–537. 10.1016/j.biocon.2006.01.015.

[ref49] Morris PA (1997) Released, rehabilitated hedgehogs: a follow-up study in Jersey. Anim Welf 6: 317–327. 10.1017/S0962728600020030.

[ref50] Möstl E, Palme R (2002) Hormones as indicators of stress. Domest Anim Endocrinol 23: 67–74. 10.1016/S0739-7240(02)00146-7. 12142227

[ref51] Mullineaux E, Pawson C (2023) Trends in admissions and outcomes at a British wildlife rehabilitation centre over a ten-year period (2012–2022). Animals (Basel) 14: 86. 10.3390/ani14010086. 38200817 PMC10778305

[ref52] Narayan E (2019) Physiological stress levels in wild koala sub-populations facing anthropogenic induced environmental trauma and disease. Sci Rep 9: 6031. 10.1038/s41598-019-42448-8. 30988329 PMC6465306

[ref53] Oliveira MM, Rodrigues DR, Araújo LMG, Leiner NO (2024) Effects of habitat quality on body condition and chronic stress in Brazilian non-volant small mammals. Anim Conserv 27: 863–873. 10.1111/acv.12972.

[ref54] Palme R (2005) Measuring fecal steroids: guidelines for practical application. Ann N Y Acad Sci 1046: 75–80. 10.1196/annals.1343.007. 16055844

[ref55] Palme R (2012) Monitoring stress hormone metabolites as a useful, non-invasive tool for welfare assessment in farm animals. Anim Welf 21: 331–337. 10.7120/09627286.21.3.331.

[ref56] Palme R (2019) Non-invasive measurement of glucocorticoids: advances and problems. Physiol Behav 199: 229–243. 10.1016/j.physbeh.2018.11.021. 30468744

[ref57] Palme R (2026) Palme-2019-PhysiolBehav-Suppl tab S1-S9-ref-updated 1^st^ April 2026. https://www.researchgate.net/publication/359740430_Palme-2019-PhysiolBehav-Suppl_Tab_S1-S9-Ref-updated_1st_July_2026 (date last accessed 19 February 2026).

[ref58] Palme R, Robia C, Baumgartner W, Möstl E (2000) Transport stress in cattle as reflected by an increase in faecal cortisol metabolite concentrations. Vet Rec 146: 108–109. 10.1136/vr.146.4.108. 10682697

[ref59] Palme R, Touma C, Arias N, Dominchin MF, Lepschy M (2013) Steroid extraction: get the best out of faecal samples. Wien Tierarztl Monatsschrift 100: 238–246.

[ref60] Pfäffle M (2015) Influence of parasites on fitness parameters of the European hedgehog (*Erinaceus europaeus*). https://www.researchgate.net/publication/275340477_Influence_of_parasites_on_fitness_parameters_of_the_European_hedgehog_Erinaceus_europaeus (date last accessed 30 April 2026).

[ref61] PfÄFfle M, Petney T, Elgas M, Skuballa J, Taraschewski H (2009) Tick-induced blood loss leads to regenerative anaemia in the European hedgehog (*Erinaceus europaeus*). Parasitology 136: 443–452. 10.1017/S0031182009005514. 19216826

[ref62] Poston JDL, Conde E, Field LM (2023) Applied Regression Models in the Social Sciences. Cambridge University Press, Cambridge.

[ref63] R Core Team (2024) A Language and Environment for Statistical Computing. R Foundation for Statistical Computing, Vienna.

[ref64] Rasmussen SL, Kalliokoski O, Dabelsteen T, Abelson K (2021) An exploratory investigation of glucocorticoids, personality and survival rates in wild and rehabilitated hedgehogs (*Erinaceus europaeus*) in Denmark. BMC Ecol 21: 1–16. 10.1186/s12862-021-01816-7. PMC8141197 34022803

[ref92] Robinson D, Couch S, and Hvitfeldt E (2026). broom: Convert Statistical Objects into Tidy Tibbles.

[ref66] Rodewald AD, Gehrt SD (2014) Wildlife population dynamics in urban landscapes. In RA McCleery, CE Moorman, MN Peterson, eds, Urban Wildlife Conservation: Theory and Practice. Springer US, Boston, pp. 117–147.

[ref67] Rothschild DM, Serfass TL, Seddon WL, Hegde L, Fritz RS (2008) Using fecal glucocorticoids to assess stress levels in captive river otters. J Wildl Manage 72: 138–142. 10.2193/2005-700.

[ref68] Sapolsky RM, Romero LM, Munck AU (2000) How do glucocorticoids influence stress responses? Integrating permissive, suppressive, stimulatory, and preparative actions. Endocr Rev 21: 55–89. 10696570 10.1210/edrv.21.1.0389

[ref69] Sauer EL, Hite JL, DuRant SE (2025) The nutritional content of anthropogenic resources affects wildlife disease dynamics. Integr Comp Biol 65: 342–353. 10.1093/icb/icaf086. 40632490

[ref70] Scott DM, Fowler R, Sanglas A, Tolhurst BA (2023) Garden scraps: agonistic interactions between hedgehogs and sympatric mammals in urban gardens. Animals (Basel) 13: 590. 10.3390/ani13040590. 36830377 PMC9951724

[ref71] Seguel M, Perez-Venegas D, Gutierrez J, Crocker DE, DeRango EJ (2019) Parasitism elicits a stress response that allocates resources for immune function in South American fur seals *(Arctocephalus australis)*. Physiol Biochem Zool 92: 326–338. 10.1086/702960. 30986114

[ref72] Shave JR, Derocher AE, Cherry SG, Thiemann GW (2019) Chronic stress and body condition of wolf-killed prey in Prince Albert National Park, Saskatchewan. Conserv Physiol 7: 1–10. 10.1093/conphys/coz037. PMC6618025 31308948

[ref73] Sheriff MJ, Dantzer B, Delehanty B, Palme R, Boonstra R (2011) Measuring stress in wildlife: techniques for quantifying glucocorticoids. Oecologia 166: 869–887. 10.1007/s00442-011-1943-y. 21344254

[ref74] Smith C, Uzal A, Warren M (2020) Statistics in R for Biodiversity Conservation, Chapter 10: Bernoulli Generalised Linear Model (GLM) 152–167 Independent publisher. ISBN-13: 9798682046942.

[ref76] South KE, Haynes K (2018) Parasitic burdening and rehabilitation of the European hedgehog, *Erinaceus europaeus*. J Wildl Rehab 38: 25–29.

[ref77] Souza L, Duarte J, Ferrari B, Rola L (2026) Fecal glucocorticoid monitoring - a valuable approach to noninvasive assessment of cortisol and stress in wild animals. In SL Nelson, ed, Wildlife Management - Perspectives From Ecology and Conservation Biology. IntechOpen, London.

[ref78] Stoks R (2001) Food stress and predator-induced stress shape developmental performance in a damselfly. Oecologia 127: 222–229. 10.1007/s004420000595. 24577653

[ref79] Touma C, Palme R (2005) Measuring fecal glucocorticoid metabolites in mammals and birds: the importance of validation. Ann N Y Acad Sci 1046: 54–74. 10.1196/annals.1343.006. 16055843

[ref80] Touma C, Sachser N, Möstl E, Palme R (2003) Effect of sex and time of day on metabolism and excretion of corticosterone in urine and feces of mice. Gen Comp Endocrinol 130: 267–278. 10.1016/s0016-6480(02)00620-2. 12606269

[ref81] Vezyrakis A, Bontzorlos V, Rallis G, Ganoti M (2023) Two decades of wildlife rehabilitation in Greece: major threats, admission trends and treatment outcomes from a prominent rehabilitation Centre. J Nat Conserv 73: 126372. 10.1016/j.jnc.2023.126372.

[ref82] Wey TW, Lin L, Patton ML, Blumstein DT (2015) Stress hormone metabolites predict overwinter survival in yellow-bellied marmots. Acta Ethol 18: 181–185. 10.1007/s10211-014-0204-6.

[ref83] White K, Hänninen L, Sainmaa S, Valros A (2024) Reasons for admission and rehabilitation rates of various wildlife species in Finland. Front Vet Sci 11: 1455632. 10.3389/fvets.2024.1455632. 39415951 PMC11479885

[ref84] Wickham H, Averick M, Bryan J, Chang W, McGowan L, François R, Grolemund G, Hayes A, Henry L, Hester J et al (2019) Welcome to the Tidyverse. J Open Source Softw 4: 43, 1686. 10.21105/joss.0168 (date last accessed 19 February 2026).

[ref85] Wilkinson L (2011) In H Wickham, ed, ggplot2: Elegant Graphics for Data Analysis Vol 67. Blackwell Publishing Inc, Malden, pp. 678–679.

[ref86] Williams BM, Baker PJ, Thomas E, Wilson G, Judge J, Yarnell RW (2018) Reduced occupancy of hedgehogs *(Erinaceus europaeus)* in rural England and Wales: the influence of habitat and an asymmetric intra-guild predator. Sci Rep 8: 12110–12156. 10.1038/s41598-018-30130-4. 30190482 PMC6127255

[ref87] Wolf TE, Valades GB, Simelane P, Bennett NC, Ganswindt A (2018) The relationship between physical injury, body condition and stress-related hormone concentrations in free-ranging giraffes. Wildl Biol 2018: 1–6. 10.2981/wlb.00460.

[ref88] Yarnell RW, Pettett CE (2020) Beneficial land management for hedgehogs *(Erinaceus europaeus)* in the United Kingdom. Animals (Basel) 10: 1566–1510. 10.3390/ani10091566. 32899181 PMC7552150

[ref89] Yarnell RW, Surgey J, Grogan A, Thompson R, Davies K, Kimbrough C, Scott DM (2019) Should rehabilitated hedgehogs be released in winter? A comparison of survival, nest use and weight change in wild and rescued animals. Eur J Wildl Res 65: 1–10. 10.1007/s10344-018-1244-4.

[ref90] Young C, Bonnell TR, Brown LR, Dostie MJ, Ganswindt A, Kienzle S, McFarland R, Henzi SP, Barrett L (2019) Climate induced stress and mortality in vervet monkeys. R Soc Open Sci 6: 191078. 10.1098/rsos.191078. 31827846 PMC6894595

[ref91] Young KM, Walker SL, Lanthier C, Waddell WT, Monfort SL, Brown JL (2004) Noninvasive monitoring of adrenocortical activity in carnivores by fecal glucocorticoid analyses. Gen Comp Endocrinol 137: 148–165. 10.1016/j.ygcen.2004.02.016. 15158127

