# Supplementary material for: Spikes in the data: biological validation and applications of an assay for detecting faecal glucocorticoid metabolites in Western European hedgehogs (Erinaceus europaeus)
Source: Conserv Physiol. 2026 Aug 24;14(1):coag063. doi: 10.1093/conphys/coag063 (PMC13500922; doi:10.1093/conphys/coag063)
Supplement: Supplementary file 1 [file web_material_coag063.zip › Supplementary Material.pdf]

Supplementary Material

ROC Analysis

The binomial Gaussian Linear Model in figure 4 was analysed further to determine the model fit. A Receiver Operator Characteristic (ROC) curve was calculated and the Area Under the Curve (AUC) of the model was 0.73 showing acceptable discrimination (Supplementary Figure 1).

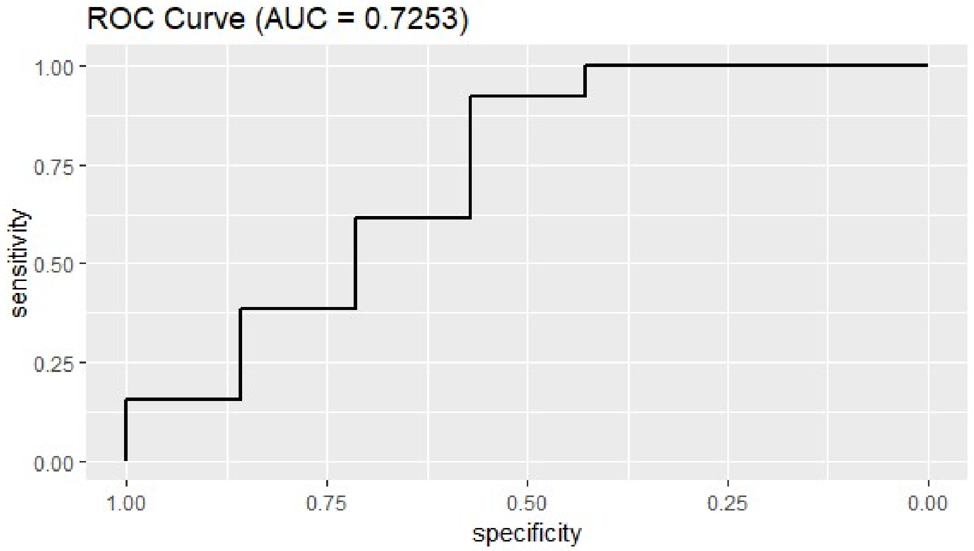

Supplementary Figure 1: Receiver Operator Characteristic (ROC) curve of a logistic regression model of outcomes for 71 Western European hedgehogs in rehabilitation by FGM concentration. Overall model fit showed a pseudo R^2=0.33. ROC curve with Area Under the Curve (AUC) = 0.73 showing acceptable discrimination. FGM concentrations reported in nanograms per gram.

Rehabilitation protocols

Wild Hogs Hedgehog Rescue is a hedgehog-only rehabilitation centre in Gloucester, UK. The centre operates to the British Wildlife Rehabilitation Council standards (British Wildlife Rehabilitation Council, 2025). Animals admitted to rehabilitation are found by members of the public or transferred from local veterinary practices. Animals that cannot be successfully rehabilitated are euthanised by a veterinarian. Animals are housed individually in a suitable enclosure (cage/hutch) and provided with torn newspaper bedding, cat food and water. They are cleaned out daily, and treatment takes place during cleaning-out to minimise handling. Animals are released as soon as possible once treatment has been completed.

Hedgehogs are admitted to rehabilitation for a range of reasons, including physical injuries (traffic-related injuries, grass cutting machinery, netting, dog bites, or being trapped), those in poor condition (underweight, malnourished, dehydrated, infected with disease or parasites) or dependent hoglets found alone following maternal deaths or nest disturbances (Martínez, Rosique and Royo, 2014; Burroughes,

Dowler and Burroughes, 2021; Vezyrakis *et al.*, 2023; Mullineaux and Pawson, 2024; White *et al.*, 2024).

All animals admitted to rehabilitation followed the same admission process, which acted as a common stressor. Animals were captured, transported, examined, injected with subcutaneous Hartmann's fluids and, where necessary, given appropriate treatment for their condition(s).

Samples from hedgehogs were only analysed for the first 7 days; therefore, stress from periods of captivity beyond the first week was not investigated in this study. The centre follows the best-practice principles of wild animal rehabilitation, and centres that do not follow these guidelines may achieve different results.

References

British Wildlife Rehabilitation Council (2025) 10 principles of wildlife care https://www.bwrc.org.uk/10-principles/

Martínez JC, Rosique AI, Royo MS (2014) Causes of admission and final dispositions of hedgehogs admitted to three wildlife rehabilitation centers in eastern Spain. *Hystrix* 25: 107–110

Burroughes ND, Dowler J, Burroughes G (2021). Admission and survival trends in hedgehogs admitted to RSPCA wildlife rehabilitation centres. *Proceedings of the Zoological Society* 74: 198–204

Vezyrakis A, Bontzorlos V, Rallis G, Ganoti M (2023) Two decades of wildlife rehabilitation in Greece: Major threats, admission trends and treatment outcomes from a prominent rehabilitation centre. *Journal for Nature Conservation* 73: 126372

Mullineaux E, Pawson C (2023) Trends in admissions and outcomes at a British wildlife rehabilitation centre over a ten-year period (2012–2022). *Animals (Basel)* 14: 86

White K, Hänninen L, Sainmaa S, Valros A (2024). Reasons for admission and rehabilitation rates of various wildlife species in Finland. *Frontiers in Veterinary Science* 11: 1455632
